# CCR9 initiates epithelial–mesenchymal transition by activating Wnt/β-catenin pathways to promote osteosarcoma metastasis

**DOI:** 10.1186/s12935-021-02320-0

**Published:** 2021-12-04

**Authors:** Haoran Kong, Wenhui Yu, Zhuning Chen, Haonan Li, Guiwen Ye, Jiacong Hong, Zhongyu Xie, Keng Chen, Yanfeng Wu, Huiyong Shen

**Affiliations:** 1 Department of Orthopedics, Sun Yat-Sen Memorial Hospital, Sun Yat-Sen University, Guangzhou, People’s Republic of China; 2 Department of Orthopedics, The Eighth Affiliated Hospital, Sun Yat-Sen University, No. 3025, Shennan Middle Road, Futian District, Shenzhen, Guangdong 518033 People’s Republic of China; 3 Center for Biotherapy, The Eighth Affiliated Hospital, Sun Yat-Sen University, No. 3025, Shennan Middle Road, Futian District, Shenzhen, Guangdong 518033 People’s Republic of China

**Keywords:** CCR9, Lung metastasis, Osteosarcoma, Epithelial–mesenchymal transition, Wnt/β-catenin

## Abstract

**Background:**

Osteosarcoma (OS) patients with lung metastasis have poor prognoses, and effective therapeutic strategies for delaying or inhibiting the spread of lung metastasis from the primary OS site are lacking. Hence, it is critical to elucidate the underlying mechanisms of OS metastasis and to identify additional new effective treatment strategies for patients.

**Methods:**

Differential expression and functional analyses were performed to identify key genes and relevant signaling pathways associated with OS lung metastasis. The expression of CCR9 in OS cell lines and tissues was measured by RT-qPCR, western blotting and immunohistochemistry. Cell migration and invasion were assessed by wound healing and Transwell Matrigel invasion assays, respectively. The regulatory relationship between CCR9 and the Wnt/β-catenin signaling pathway was further evaluated by rescue experiments.

**Results:**

The expression of CCR9 was elevated in OS cell lines and patients with lung metastasis. CCR9 promoted MG63 and HOS cell migration and invasion by activating the Wnt/β-catenin signaling pathway. Furthermore, knockdown of CCR9 repressed epithelial–mesenchymal transition (EMT) by downregulating mesenchymal markers (N-cadherin and Vimentin) and EMT-associated transcription factors (twist and snail) and upregulating an epithelial marker (E-cadherin).

**Conclusions:**

Our findings suggest that CCR9 promotes EMT by activating Wnt/β-catenin pathways to promote OS metastasis. CCR9 may be a promising therapeutic target to inhibit lung metastasis and serve as a novel prognostic marker for OS.

**Supplementary Information:**

The online version contains supplementary material available at 10.1186/s12935-021-02320-0.

## Background

Osteosarcoma (OS) is the most common primary sarcoma of bone and is diagnosed primarily in adolescents and young adults [1]. The metaphyses of long bones, the most active sites in bone, are major sites of OS growth [2]. OS is characterized by local aggressive growth and high metastatic potential; the lung is the most common site of metastasis, and lung metastasis is the main cause of death from OS [3]. In patients with inoperable OS, the median time to progression is approximately 1.8 months, with a median survival of approximately 6 months and a 2-year survival of < 2% [4]. Currently, the main treatment options for OS include neoadjuvant chemotherapy, tumor resection and postoperative chemotherapy. The most commonly used chemotherapy drugs in OS treatment are doxorubicin, cisplatin and methotrexate [5]. These treatments have greatly increased the survival rate of OS patients without metastasis over the past forty years [6]. However, although numerous novel therapies have been developed, the prognosis of patients with lung metastasis has not significantly improved, and the 5-year survival rate is at most 30% [7]. Lung metastasis is the primary challenge for OS therapy, but effective therapeutic strategies for delaying or inhibiting the spread of lung metastasis from the primary OS site are lacking. Hence, it is critical to elucidate the underlying mechanisms of OS metastasis and to identify additional new effective treatment strategies for patients.

CCR9 is located on chromosome 3p21.31 and belongs to the β-chemokine receptor family. It is mainly distributed in immature T lymphocytes and on the surface of intestinal cells, where it plays a crucial role in T lymphocyte development and tissue‐specific homing after binding to its specific ligand, CCL25 [8]. Binding of CCR9 to its ligand regulates diverse physiological and pathological processes, including cell growth, tissue injury, inflammatory reactions, angiogenesis, and tumorigenesis [9, 10]. Some previous studies have shown that CCR9 is overexpressed in a variety of tumors and plays key roles in tumorigenesis and cancer progression [11–13]. Moreover, recent studies have demonstrated that CCR9 can regulate many signaling pathways in cancers, especially pathways involved in tumor metastasis and chemotherapeutic resistance [14–16]. However, the expression and effect of CCR9 in OS have not been investigated in detail.

EMT is a biological process characterized by downregulation of epithelial markers (e.g., E‐cadherin) and upregulation of mesenchymal markers (e.g., N‐cadherin and Vimentin) [14]. In the initial phase of metastasis, cancer cells undergo EMT, during which they lose their epithelial characteristics to acquire mesenchymal polarity with a high capability for mobility, and metalloproteinases (MMPs) are upregulated to degrade the extracellular matrix (ECM); these events collectively result in the acquisition of invasive properties [17]. EMT is considered the crucial mechanism associated with cancer metastasis. During the metastasis and invasion of cancer cells, the EMT program can be activated by upstream transcription factors, such as twist, snail, slug and ZEB [18]. Recently, various studies have revealed that EMT occurs in some nonepithelial tumor types, such as OS, and that it is related to metastasis and invasion in OS [19]. Abnormal expression of EMT-inducing transcription factors has also been observed in OS [20]. However, the specific roles and molecular mechanism of these transcription factors in OS are less defined.

In this study, differentially expressed genes (DEGs) were screened based on TCGA data, and a protein–protein interaction (PPI) network was then constructed. We found that CCR9 is a key hub gene and may play critical roles in OS metastasis. This paper is the first to provide evidence that CCR9 promotes migration and invasion by activating the Wnt/β-catenin pathway in OS cells. Furthermore, we demonstrated that knockdown of CCR9 attenuates EMT in OS. Our findings suggest that CCR9 may be a therapeutic target for lung metastasis of OS.

## Methods

### Bioinformatics analysis

RNA sequencing data for OS patients were downloaded from the TCGA website (https://tcga-data.nci.nih.gov/tcga/). The characteristics of the patients are shown in Table 1. The patients were divided into two groups according to the absence or presence of lung metastasis. DEGs between the two groups were selected using DESeq2, and DEGs with p-values < 0.05 and |log2 (fold change)| values > 1 were selected for further analyses. To characterize the functional roles of the above DEGs, Gene Ontology (GO) functional enrichment and Kyoto Encyclopedia of Genes and Genomes (KEGG) pathway enrichment analyses were performed using the Database for Annotation, Visualization and Integrated Discovery (DAVID) Bioinformatics Resources 6. (https://david.ncifcrf.gov/). The Search Tool for the Retrieval of Interacting Genes/Proteins (STRING) v11 was used to construct the PPI network (PPI combined score > 0.7) and identify key hub genes in the PPI network [21]. The correlation of key hub genes in the modules was analyzed with Cytoscape v3.7.1. Gene set enrichment analysis (GSEA) was conducted by using Gene Set Enrichment Analysis software and methods (http://www.broadinstitute.org/gsea) [22].

**Table1:** Clinical features of OS patients with or without lung metastasis

Clinical features	OS patients with lung metastasis	OS patients without lung metastasis	P value
	(n, %)	(n, %)	
All patients	22(100)	65(100)	
Age at diagnosis, year			
≤ 18	19(86.4)	54(83.1)	0.7169
> 18	3(13.6)	11(16.9)	
Gender			
Male	10(45.5)	40(61.5)	0.1872
Female	12(54.5)	25(38.5)	
Tumor site			
Femur	14(63.7)	26(40.0)	0.2483
Tibia	3(13.6)	18(27.7)	
Fibula	1(4.5)	7(10.8)	
Others	4(18.2)	14(21.5)	
Relapse			
Yes	14(63.6)	24(36.9)	0.0290*
No	8(36.4)	41(63.1)	

^*^Statistically significant

### Cell lines, reagents and antibodies

Bone marrow-derived mesenchymal stem cells (BM-MSCs) were isolated and cultured as previously described [23]. The human OS cell lines MG63, Saos2, HOS, and U2OS were purchased from the American Type Culture Collection (ATCC). BM-MSCs and human OS cell lines were cultured in DMEM (CR-12800, Cienry) supplemented with 10% FBS (11011-8611, Hangzhou Sijiqing Co., Ltd), penicillin (50 U/mL; Gibco), and streptomycin (50 µg/mL; Gibco), and cells were maintained in humidified 37 °C incubators with 5% CO_2_. Antibodies against CCR9 (ab32556), total β-catenin (ab68183), active β-catenin (ab246504), and GAPDH (ab9485) were purchased from Abcam. Antibodies against E-cadherin (sc-8426), N-cadherin (sc-59987), vimentin (sc-6260), Twist (sc-81417), Snail (sc-271977), and MMP-1 (sc-21731) were obtained from Santa Cruz Biotechnology. Primary antibodies were used for western blotting at a dilution of 1:1000, and secondary antibodies were used at a dilution of 1:3000. The primary antibodies and the secondary antibodies for immunofluorescence were used at dilutions of 1:400 and 1:1000. The pathway inhibitor XAV-939 (HY-15147, Med Chem Express, Inc.) was used to inhibit the Wnt/β-catenin signaling pathway as previously described [24].

### Cell transfection

The CCR9 overexpression plasmid, specific small interfering RNA (siRNA) targeting CCR9 (si-CCR9) and scrambled control siRNA (si-NC) were purchased from Guangzhou IGE Biotechnology Ltd. Next, MG63 and HOS cells were chosen for subsequent transfection. All transfection experiments were performed using Lipofectamine^®^ RNAiMAX Reagent (L3000008, Thermo Fisher Scientific) according to the manufacturer’s instructions.

### RNA extraction and RT-qPCR

Total RNA from cell lines and frozen tumor tissues was isolated using an RNA-Quick Purification Kit (ES-RN001, Shanghai Yishan Biotechnology Co., Ltd.). cDNA was synthesized using Evo M-MLV RT Premix for qPCR (AG11706, Accurate Biotechnology). qPCR was performed using a SYBR^®^ Green Premix Pro Taq HS qPCR Kit (AG11701, Accurate Biotechnology) in a Roche LightCycler 480 qPCR System (Roche, Switzerland). Relative gene expression levels were calculated by normalization to GAPDH expression using the 2 − ΔΔCt method. All primer sequences are provided in Additional file 1: Table S1.

### Western blot analysis

MG63 and HOS cells were seeded in 12-well plates (50,000 cells/well) and subjected to different treatments. Then, total protein was extracted using a mixture of phenylmethylsulfonyl fluoride (PMSF, 36978, Thermo Fisher Scientific) and radioimmunoprecipitation assay (RIPA) lysis buffer (89900, Thermo Fisher Scientific). The total protein of the fresh frozen OS tissue with or without lung metastasis was extracted using the Tissue Protein Extraction Reagent (78510, Thermo Fisher Scientific). Western blot analysis was performed using standard procedures. For all samples, expression levels were normalized to those of GAPDH.

### Cell proliferation assays

Cell counting kit-8 (CCK-8) and colony formation assays were performed to test cell proliferation. In the CCK-8 assay, 2 × 10^3^ cells/well were seeded in 96-well plates and incubated with CCK-8 reagent (C0038, Beyotime) at 37 °C for another 2 h. For the colony formation assay, 1 × 10^3^ cells/well were cultured in a 6-well plate. The medium was changed every 3 days, and cell growth was observed. After 10 days or when the number of cells per colony exceeded 50, the cells were fixed with 4% paraformaldehyde (P0099, Beyotime) and stained with 1% crystal violet solution (C0121, Beyotime).

### Flow cytometry

Flow cytometric analyses were performed to evaluate apoptosis with a PE Annexin V Apoptosis Detection Kit I (559783, BD Pharmingen). A flow cytometer (BD Biosciences) was used to detect apoptosis, and the data were analyzed with FlowJo v10 software.

### Wound healing assay

The cell migration capacity was evaluated with a wound healing assay. A total of 1 × 10^5^ cells were seeded in 12-well plates in 2 ml of medium and incubated at 37 °C in 5% CO_2_. The cell layer was scratched with a sterile 10 μl pipette tip at a confluence of approximately 90%. The floating cells were washed out with PBS buffer, and the adherent cells were then cultured in serum-free medium. The wound healing assay images were taken at 0 and 24 h with a Nikon microscope. And in the overexpression experiment, images were acquired at 0 and 18 h. The area of wound healing was quantified with ImageJ software.

### Transwell migration assay and matrigel invasion assay

The migration and invasion capacities of MG63 and HOS cells were evaluated in 24-well plates with Transwell inserts (8-µm pore size, Corning) without a Matrigel coating for the migration assay and with a Matrigel coating for the invasion assay. MG63 and HOS cells (1 × 105/ml) in 200 µl of serum-free medium were added to the upper chambers, and 600 µl of 10% FBS medium was placed in the lower chambers. Cell migration and invasion were allowed to proceed for 24 h at 37 °C in 5% CO_2_. The cells that migrated into the lower chamber or invaded the lower surface of the membranes were stained with crystal violet (C0121, Beyotime) for 30 min. Cells were counted with an upright microscope. The average numbers of migrated and invaded cells were determined by counting the cells in six random high-power fields (200×).

### Immunohistochemistry

All 31 OS tissue samples (21 without lung metastasis and 10 with lung metastasis) are from patients hospitalized in the Sun Yat-sen Memorial Hospital of Sun Yat-sen University and The Eighth Affiliated Hospital of Sun Yat-sen University and between December 2016 and December 2020. None of the patients underwent chemotherapy or radiotherapy before surgery. All the OS tissue samples with lung metastasis were classified TNM stage IV. The numbers of OS patients without lung metastasis classified by TNM stage were as follows: 5 were at TNM stage I; 10 were at TNM stage II and 6 at TNM stage III. Immunohistochemistry (IHC) analysis of CCR9 expression in OS tissues with or without lung metastasis was performed according to standard protocols. In brief, the OS tissue microarray was deparaffinized, rehydrated, and washed, antigen retrieval was performed with citrate antigen retrieval solution, and endogenous peroxidase activity was blocked. Then, the array was incubated first with an anti‐CCR9 primary antibody overnight and then with a biotinylated secondary antibody for 1 h. Subsequently, the array was stained with diaminobenzidine and hematoxylin. The histological scores (H-score) were evaluated and defined by experienced pathologists in a blinded manner as previously described [25]. Briefly, the H-score was calculated by the proportion score multiplied by the staining intensity score. The staining intensity was graded as 0, negative staining; 1, weak staining; 2, moderate staining; and 3, strong staining. The proportion of positively stained cells per sample was determined as follows: 0 for < 5%; 1 for 6–25%; 2 for 26–50%; 3 for 51–75%; and 4 for 76–100% of the examined cells. Then, the array was observed under a microscope.

### Statistical analysis

All in vitro experiments were performed at least in triplicate, and data are presented as the mean ± SD values. All statistical analyses were performed by using GraphPad Prism version 8.0 (GraphPad Software, CA) and SPSS 26.0 software (SPSS Inc). Differences between two groups were assessed using Student’s *t* test. A P value < 0.05 was considered statistically significant.

## Results

### Identification and functional enrichment analysis of DEGs

**Fig. 1:**
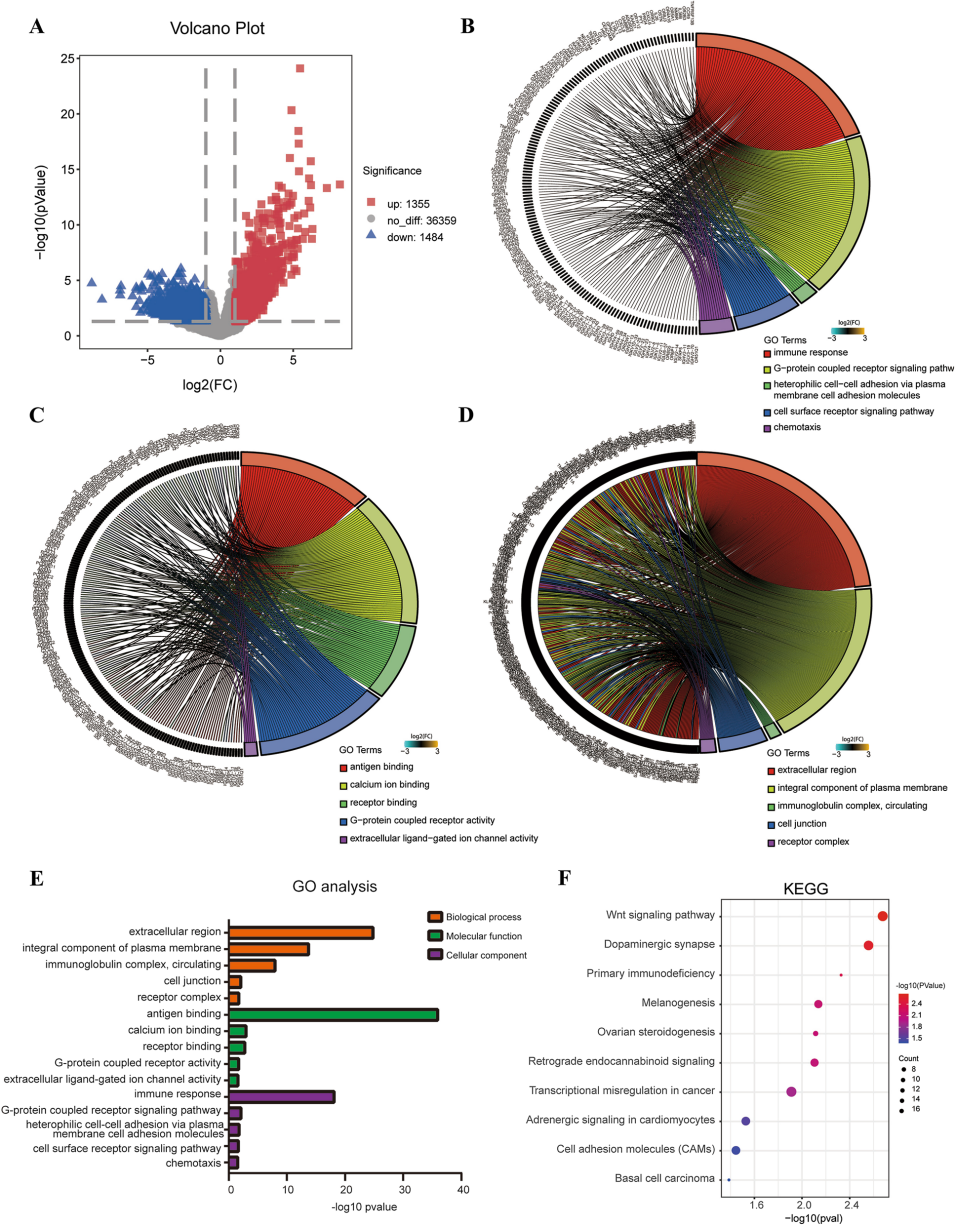
**a** Volcano plot of DEGs, including 1355 upregulated genes and 1484 downregulated genes. The threshold for the X axis was Log2fc ≥ 1 and Log2fc ≤ -1, and the threshold for the Y axis was P-value < 0.05. **b** GO enrichment analysis of DEGs revealed enriched biological process terms. **c** GO enrichment analysis of DEGs revealed enriched cellular component terms. **d** GO enrichment analysis of DEGs revealed enriched molecular function terms. **e** Top 5 enriched terms in the BP, MF and CC categories. **f** The top ten KEGG signaling pathways (KEGG analysis) enriched with the DEGs

First, transcriptome sequencing data of OS patients (22 with lung metastasis and 65 without metastasis) from the TCGA database were compared. We identified 2839 DEGs, including 1355 upregulated genes and 1484 downregulated genes, in the lung metastasis group (Fig. 1a). To explore the functional roles of the above DEGs, we performed GO and KEGG pathway enrichment analyses. These DEGs were enriched in biological process (BP), molecular function (MF), and cellular component (CC) terms. In the BP category, these DEGs were enriched in the terms immune response, G-protein coupled receptor signaling pathway, heterophilic cell–cell adhesion via plasma membrane cell adhesion molecules, cell surface receptor signaling pathway and chemotaxis (Fig. 1b). In addition, in the MF category, the GO terms most significantly enriched with the DEGs were antigen binding, calcium ion binding, receptor binding, G-protein coupled receptor activity and extracellular ligand-gated ion channel activity (Fig. 1c). In the CC category, extracellular region, integral component of plasma membrane, immunoglobulin complex circulating, cell junction, and receptor complex were the GO terms most significantly enriched with the DEGs (Fig. 1d). The top five enriched functional terms were ranked by enrichment significance (Fig. 1e). KEGG enrichment analysis showed that the DEGs were enriched mainly in the Wnt signaling pathway, dopaminergic synapse, primary immunodeficiency, and melanogenesis and ovarian steroidogenesis pathways (Fig. 1f).

### CCR9 is upregulated in human OS cell lines and OS tissues with lung metastasis

**Fig. 2:**
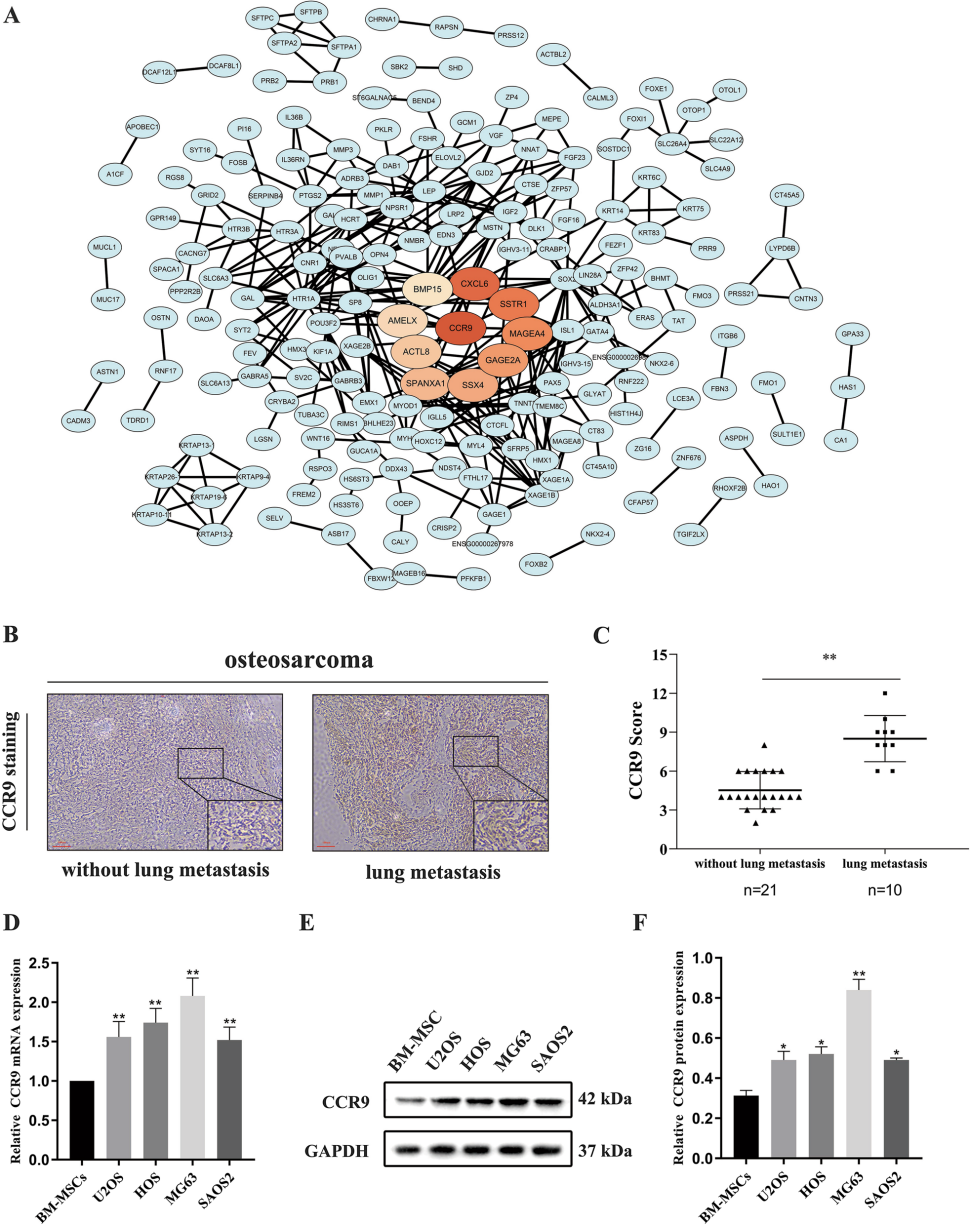
**a** PPI network of DEGs and a core module of the PPI network. CCR9 was the highest-ranked PPI hub gene. **b** Immunohistochemistry staining for CCR9 in OS tissues with or without lung metastasis. **c** CCR9 immunohistochemistry intensity score in OS tissues (21 without lung metastasis and 10 with lung metastasis), and the score was higher in OS tissues with lung metastasis. **d** Relative CCR9 mRNA expression levels in HD-MSCs, U2OS, HOS, MG63 and SAOS2 cell lines. The expression of CCR9 was higher in OS cell lines. **e** Protein expression levels of CCR9 in HD-MSCs, U2OS, HOS, MG63 and SAOS2 cell lines. **f** Quantitative western blot data for CCR9 protein expression. The expression of CCR9 was higher in OS cell lines. Mean ± SD from three independent experiments. *P < 0.05; **P < 0.01; ***P < 0.001

To better understand the interactions among proteins, we constructed the PPI network of the DEGs and rated the DEGs by using the cytoHubba plugin in Cytoscape. The PPI network of the DEGs contained 198 nodes and 334 edges. The hub genes identified to have the ten highest node degrees in the intersected PPI network were CCR9, CXCL6, SSTR1, MAGEA4, GAGE2A, SSX4, SPANXA1, ACTL8, AMELX and BMP15 (Fig. 2a). Additionally, CCR9 was identified as the highest-ranked PPI hub gene. To explore whether CCR9 is related to lung metastasis of OS, we first performed immunohistochemistry staining to examine the expression of CCR9 in 31 OS tissues (21 without lung metastasis and 10 with lung metastasis). As the immunohistochemistry results showed, CCR9 expression was higher in OS tissues from patients with lung metastasis than in OS tissues from patients without lung metastasis (Fig. 2b and c). To determine the expression of CCR9 in human OS cells, we performed qRT-PCR in OS cell lines. The qRT-PCR results showed that CCR9 was upregulated in four human OS cell lines compared with BM-MSCs (Fig. 2d). Similar results were observed for the protein level of CCR9 (Fig. 2e and f).

### Knockdown of CCR9 does not significantly impact the proliferation and apoptosis of MG63 and HOS cells

**Fig. 3:**
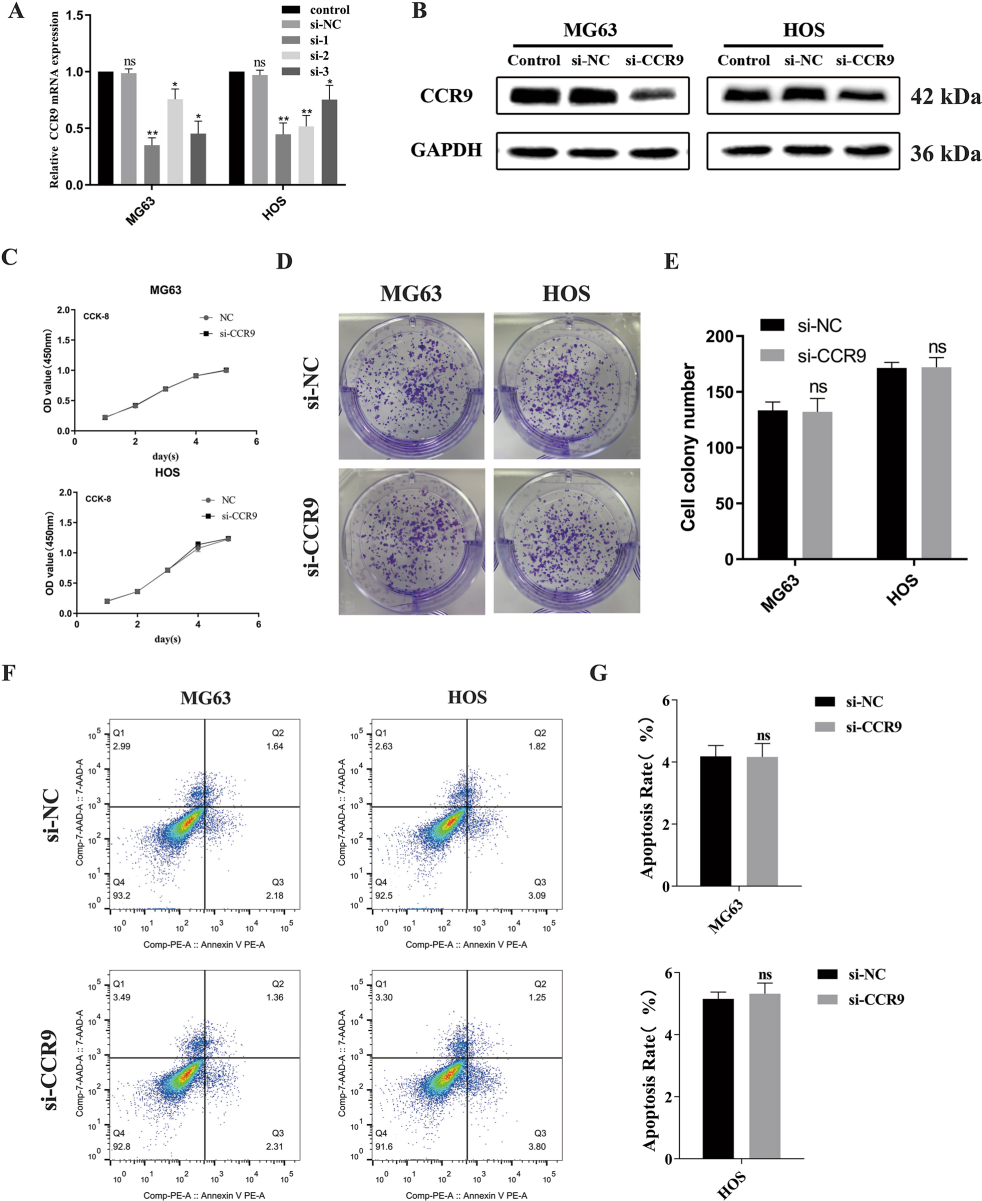
**a** MG63 and HOS cells were transfected with blank control, negative control siRNA (NC) or CCR9 siRNA (si-1, si-2 and si-3). The relative CCR9 mRNA expression levels were obviously lower in si-CCR9 group. **b** Protein expression levels of CCR9 in MG63 and HOS cells treated with either blank control, si-NC or si-CCR9, as determined by western blot analysis. The protein expression levels were apparently lower in si-CCR9 group. **c** CCK8 assay results showed no significant difference in two groups. **d** Colony formation capacity of MG63 and HOS cells, as assessed by a colony formation assay after 10 days. **e** Quantification of the cell colony number, and the results showed no significant difference in two groups. **f** The apoptosis rates of MG63 and HOS cells were analyzed by Annexin V-FITC/PI staining. The percentage of apoptotic cells was calculated as the sum of the early apoptotic (Q3) and late apoptotic cells (Q2) percentages. **g** Quantitative apoptosis rates of MG63 and HOS cells, and no significant difference in two groups. Mean ± SD from three independent experiments. *P < 0.05; **P < 0.01; ***P < 0.001

We performed a knockdown experiment with MG63 and HOS cells. Three specific siRNAs (si-1, si-2 and si-3) were transfected into MG63 and HOS cells, and the expression of CCR9 was measured by qRT-PCR and western blotting. The CCR9 mRNA level in the si-CCR9 group was significantly decreased compared to that in the control and negative control (NC) groups (Fig. 3a). Then, we selected siRNA-1, which had the highest knockdown efficiency, for subsequent experiments. The western blot results showed that the protein expression of CCR9 was downregulated in the siRNA-1 group (Fig. 3b). We then performed CCK‐8 and colony formation assays to evaluate the effect of CCR9 on OS cell proliferation. The assay demonstrated that CCR9 knockdown did not affect the proliferation ability of MG63 and HOS cells (Fig. 3c), and the colony formation assay showed similar results (Fig. 3d and e). Moreover, after knockdown of CCR9, no significant changes in apoptosis were observed in either of the two groups (Fig. 3f and g).

### Knockdown of CCR9 inhibits the migration and invasion of MG63 and HOS cells

**Fig. 4:**
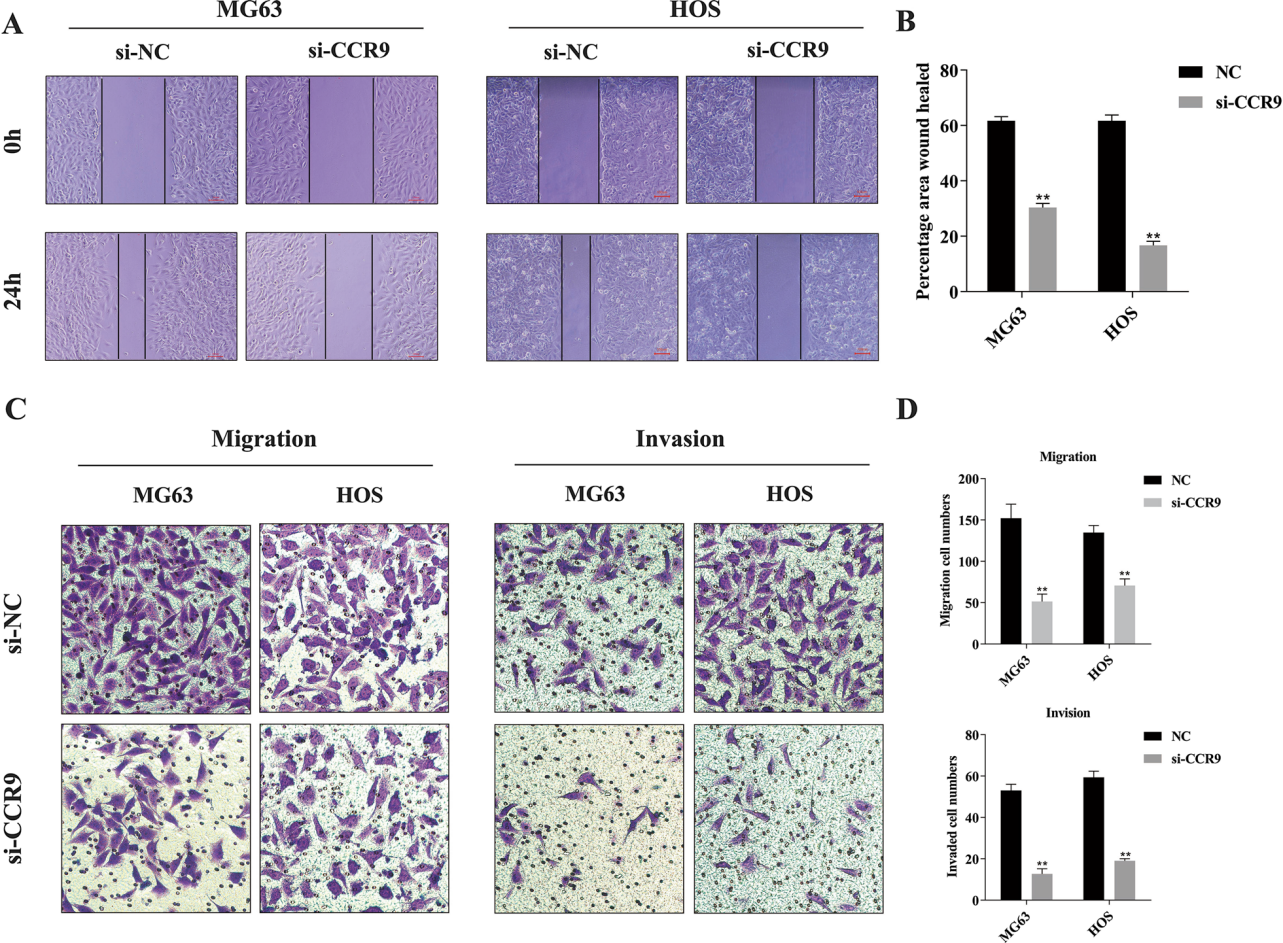
**a** The migration of MG63 and HOS cells was evaluated by a wound healing assay, and the si-CCR9 group showed slower wound healing. **b** Quantification of the percentage area of wound healing. **c** Transwell migration and invasion assays of MG63 and HOS cells, and knockdown of CCR9 significantly inhibited cell invasion. **d** Quantitative results of Transwell migration and invasion assays. Mean ± SD from three independent experiments. *P < 0.05; **P < 0.01; ***P < 0.001

To explore whether CCR9 plays a role in OS cell migration and invasion, we performed wound healing, migration and Matrigel invasion assays. The wound healing assay demonstrated that CCR9 knockdown slowed wound healing (Fig. 4a and b). Similar results were obtained for cell migration in the Transwell migration assay (Fig. 4c and d). Cell invasion activity was evaluated by a Matrigel invasion assay, which revealed that knockdown of CCR9 significantly inhibited cell invasion (Fig. 4c and d).

### Knockdown of CCR9 can reverse the progression of EMT

**Fig. 5:**
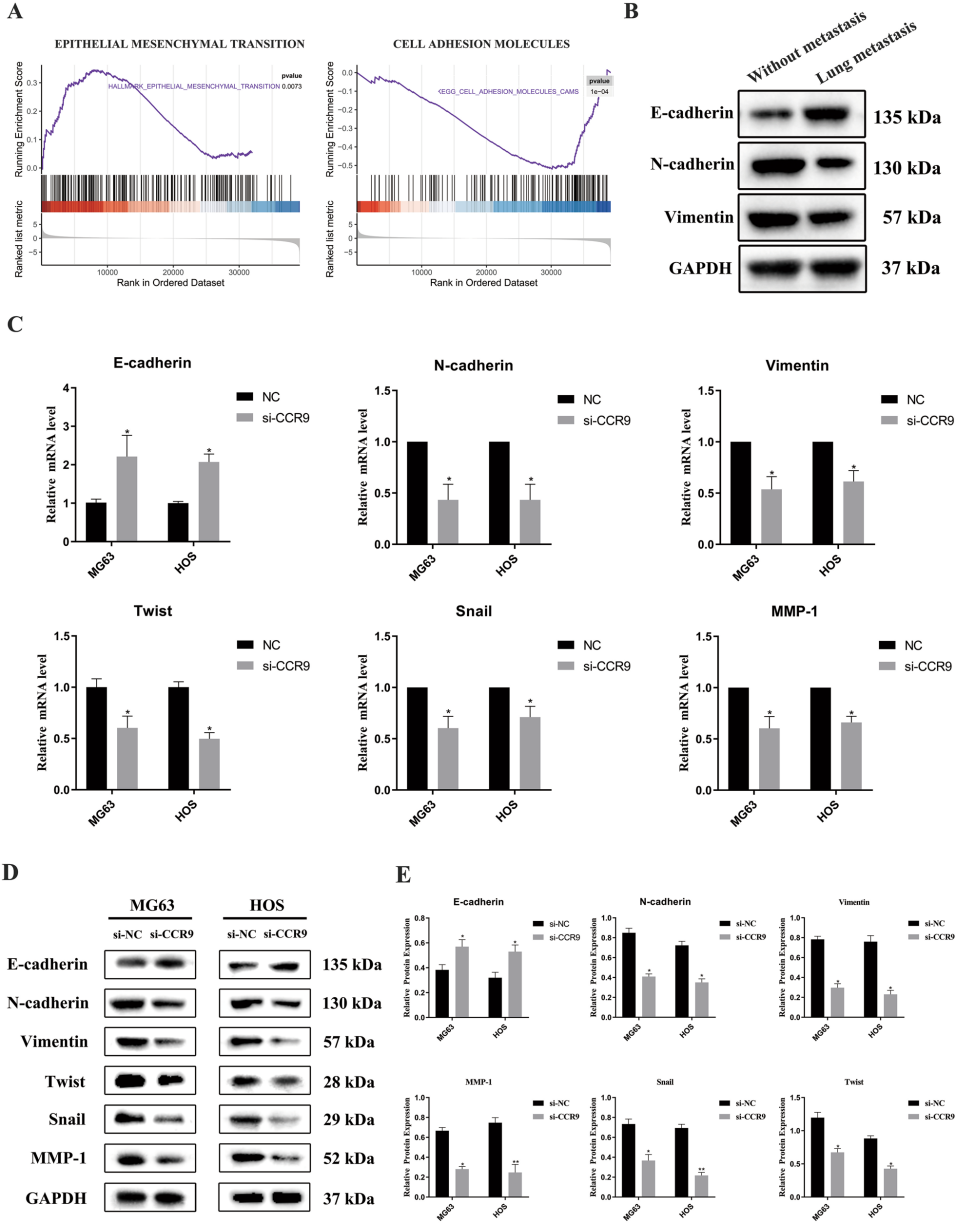
**a** The GSEA results showed significant enrichment of the gene signature associated with EMT and cell adhesion molecules. **b** The protein expression level of E-cadherin was higher in OS tissues with lung metastasis, and the expression of N-cadherin and Vimentin was downregulated. **c** The expression N-cadherin, vimentin, twist, snail and MMP-1, was obviously downregulated, and E-cadherin expression was significantly upregulated in si-CCR9 group. **d** The protein expression levels of EMT-related markers in MG63 and HOS cells. **e** Quantitative data for the protein expression levels of EMT-related markers. Mean ± SD from three independent experiments. *P < 0.05; **P < 0.01; ***P < 0.001

To identify the pathway involved in OS metastasis, we performed GSEA with genome-wide expression profiles from TCGA. The GSEA results demonstrated significant enrichment of the gene signature associated with EMT and cell adhesion molecules (Fig. 5a). To further confirm whether EMT is involved in this metastatic process, we evaluated the expression of EMT-associated markers by qRT-PCR and western blot analysis. Western blotting revealed that the expression of E-cadherin was reduced in OS tissues without lung metastasis compared with the lung metastasis group, and the expression of N-cadherin and vimentin was increased (Fig. 5b). In the CCR9 knockdown group, the expression of EMT-associated markers, such as N-cadherin, vimentin, twist, snail and MMP-1, was obviously downregulated, and E-cadherin expression was significantly upregulated (Fig. 5c). Moreover, the protein levels also showed similar changes (Fig. 5d and e).

### CCR9 promotes cell migration and invasion by activating the Wnt/β-catenin signaling pathway in OS cells

**Fig. 6:**
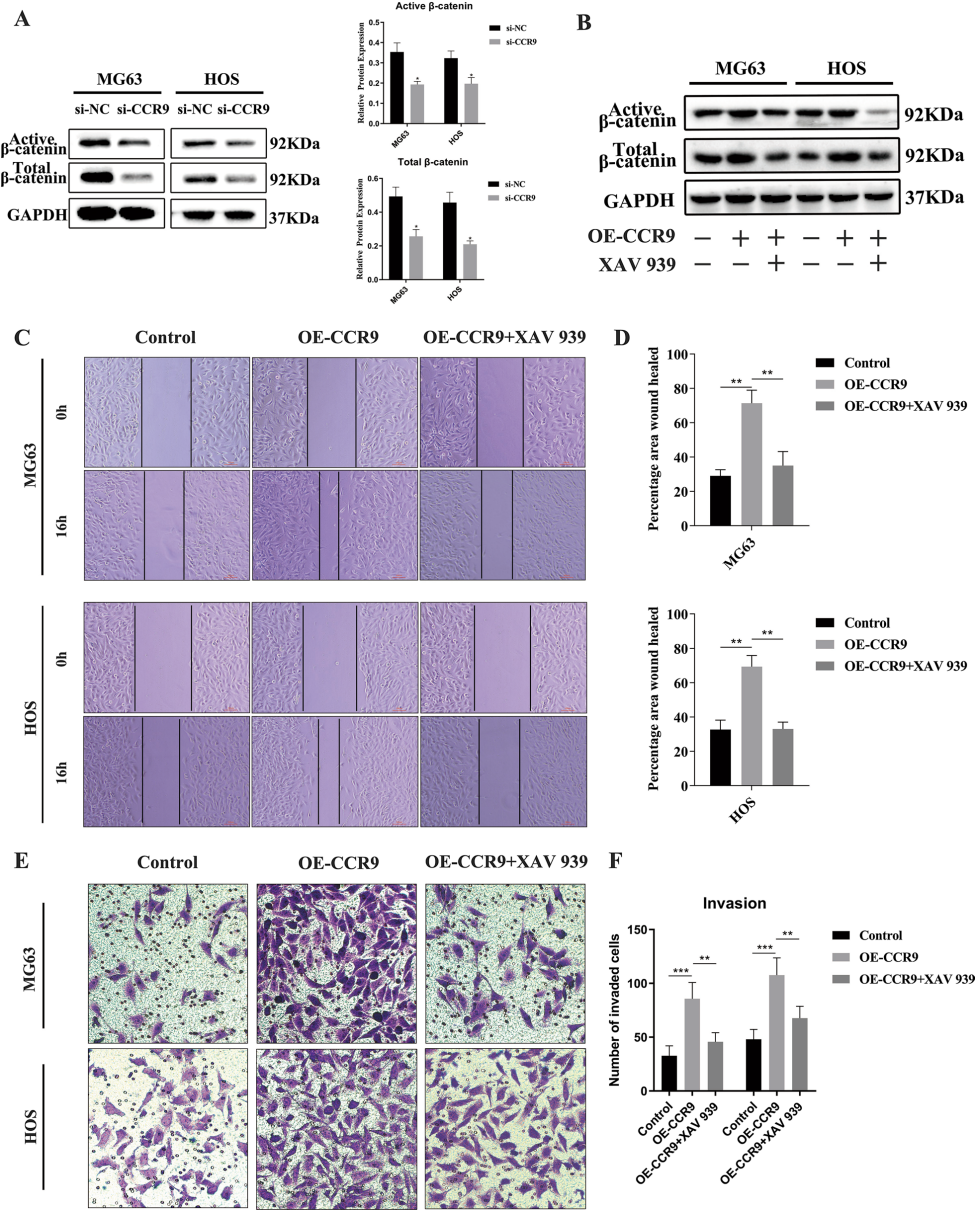
**a** The protein expression levels of active β-catenin and total β-catenin were downregulated in si-CCR9 group. **b** The protein expression levels and quantitative data of active β-catenin and total β-catenin in MG63 and HOS cells were shown. Overexpression of CCR9 activated the Wnt/β-catenin pathway, and the Wnt signaling inhibitor XAV-939 counteracted the activation. **c** Cell migration assay of MG63 and HOS cells. Overexpression of CCR9 increased the wound healing rate and the inhibitor of XAV-939 inhibited this effect. **d** Quantification of the area percentage of wound healing. **e** Transwell invasion assay of MG63 and HOS cells. Overexpression of CCR9 promoted the invasion ability and the inhibitor of XAV-939 inhibited this effect. **f** Quantitative results of the Transwell migration and invasion assays. Mean ± SD from three independent experiments. *P < 0.05; **P < 0.01; ***P < 0.001

**Fig. 7:**
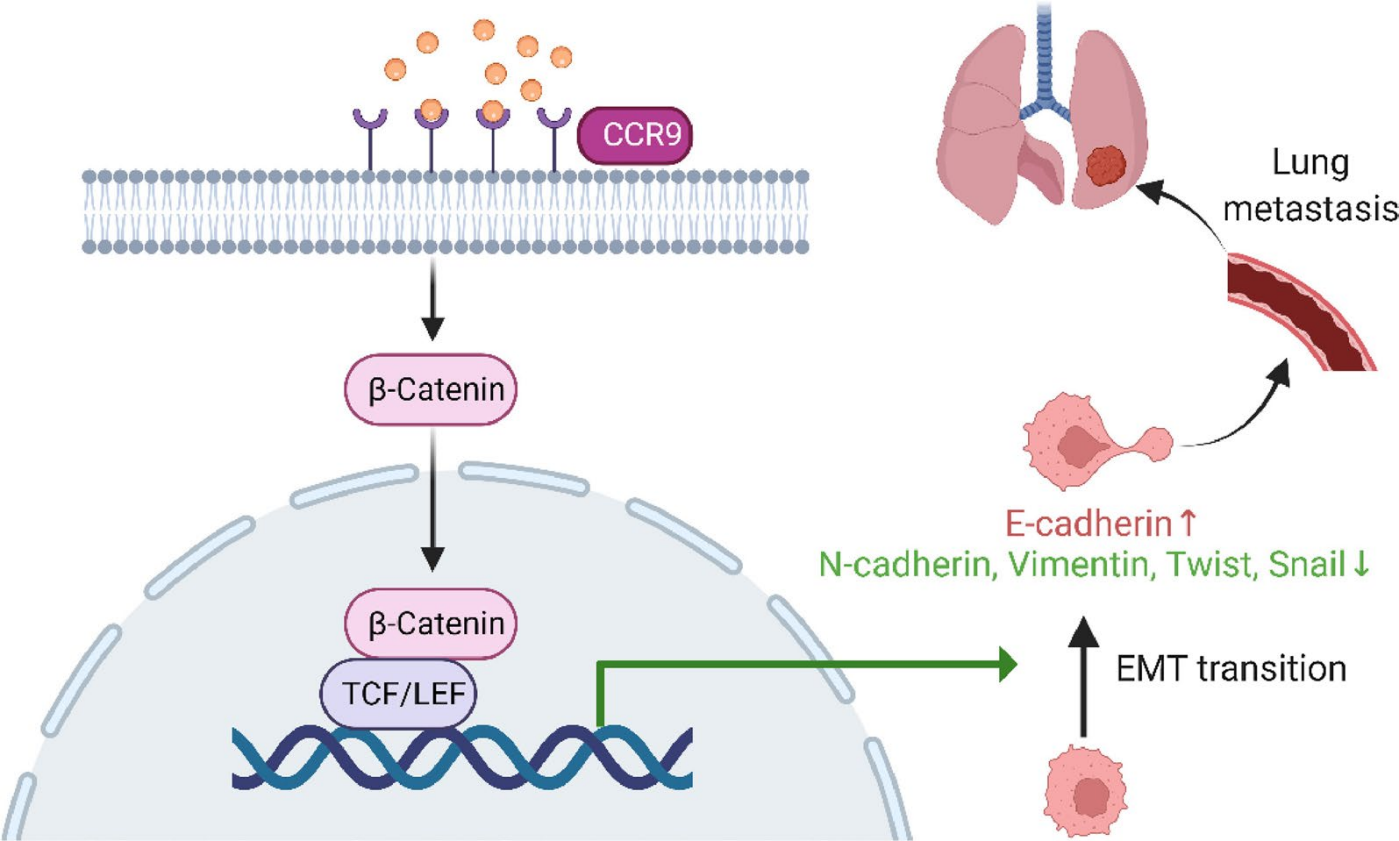
**a** Hypothesis diagram of the mechanism by which CCR9 works

Considering the results of KEGG enrichment analysis, to explore whether the Wnt/β-catenin pathway is involved in the migration and invasion of OS cells, we performed western blotting to detect the expression of total β-catenin and active β-catenin. Knockdown of CCR9 decreased the expression of both active β-catenin and total β-catenin (Fig. 6a and b). First, experiments with the Wnt signaling inhibitor XAV-939 showed that XAV-939 counteracted the activation of the Wnt/β-catenin pathway by overexpression of CCR9 (Fig. 6c). We further explored whether inhibition of the Wnt/β-catenin pathway can attenuate the migration and invasion abilities of OS cells. The wound healing assay showed that overexpression of CCR9 (OE-CCR9) increased the wound healing rate compared with that in the control group; in contrast, wound healing was significantly slower in the group of OS cells treated with OE-CCR9 and the Wnt/β-catenin signaling pathway inhibitor XAV-939 (10 µM) (Fig. 6c and d). The Transwell Matrigel ssay showed similar results (Figs. 6e and f, 7).

## Discussion

OS, the most common primary bone tumor, occurs mainly in children and adolescents [26]. Approximately 15–20% of OS patients experience pulmonary metastasis, which is the leading cause of death in OS patients [27]. Effective treatments for pulmonary metastasis of OS are lacking; thus, understanding the mechanisms involved in OS metastasis is crucial. In this study, we are the first to classify OS patients with and without lung metastasis by using RNA sequencing data from TCGA. Integrated bioinformatic analysis was performed to explore factors associated with OS lung metastasis. We found that CCR9 can promote OS cell migration and invasion by activating Wnt/β-catenin signaling pathways. Furthermore, knockdown of CCR9 can inhibit EMT by downregulating mesenchymal markers (N-cadherin and Vimentin) and EMT-associated transcription factors (twist and snail) and upregulating an epithelial marker (E-cadherin).

In our present study, an integrated analysis of RNA sequencing data of OS from TCGA was conducted, and 2839 DEGs—1355 upregulated genes and 1484 downregulated genes—were identified between OS tissues from patients with lung metastasis and patients without lung metastasis. GO enrichment analysis demonstrated that the DEGs were involved mainly in receptor activity, specifically, the terms G-protein coupled receptor activity, receptor complex and cell surface receptor signaling pathway. In addition, the DEGs were enriched in the terms cell junction and heterophilic cell–cell adhesion via plasma membrane cell adhesion molecules. Receptor activity, especially that of chemokines and their receptors, is crucial in regulating the progression and metastasis of tumors [28]. Research has shown that tight cell–cell junctions can maintain cell adhesion and prevent the metastasis of tumor cells. The above results suggested that DEGs associated with receptor activity and cell–cell junctions may be crucial in lung metastasis of OS.

CCR9, which belongs to the chemokine receptor family, has been found to be expressed in tumors and to be closely associated with tumor proliferation, apoptosis, and metastasis [8]. However, the effect of CCR9 in OS cells remains uncertain. Our PPI network analysis showed that CCR9 had the highest degree in the network. Through bioinformatic analysis, we found that CCR9 was upregulated in patients with metastatic OS and that as a key hub gene, it may play a critical role in the progression of OS metastasis. Our experiments indicated that CCR9 expression in the primary tumor site was higher in OS patients with lung metastasis than in those without lung metastasis. A previous study showed that CCR9 induces tumor migration via loss of PTEN in T-lineage acute lymphoblastic leukemia models [29]. Lu et al. [14] demonstrated that CCR9/CCL25 promotes the migration and invasion of lung adenocarcinoma cancer stem cells. Previous studies revealed that CCR9 can promote metastasis by activating different signaling pathways in tumors. However, the specific mechanisms are complex and require further research. We found that knockdown of CCR9 inhibited the migration and invasion of OS cells. Evidence indicates that CCR9 may contribute to the migration and invasion of OS. Moreover, our experimental results were consistent with the functional enrichment analysis results.

The GSEA results revealed significant enrichment of the gene signature associated with EMT and CAMS. We experimentally demonstrated that knockdown of CCR9 inhibited EMT by downregulating the N-cadherin and Vimentin expression and upregulating the E-cadherin expression in OS cells. Moreover, we revealed that knockdown of CCR9 significantly suppressed the expression of EMT-inducing transcription factors (twist and snail). In this study, we also observed that MMP-1 proteins were significantly downregulated when EMT was suppressed by knockdown of CCR9. EMT is a critical mechanism that results in tumor metastasis [30]. Many CAMs have also been implicated in the metastasis of cancer cells [31]. Notably, E-cadherin and N-cadherin are important molecular markers of EMT and belong to the CAM family. Previous studies revealed that downregulation of E-cadherin and upregulation of N-cadherin induce EMT and promote cancer cell metastasis [32]. In addition, recent studies have indicated that CCR9 can regulate EMT-associated markers to influence the progression of cancer cells; the effects observed in other cancers include decreasing the expression of E‐cadherin, increasing the expression of N‐cadherin, and significantly upregulating the expression of MMP‐1 [33]. Therefore, these studies indicate that CCR9 can induce EMT to affect OS metastasis.

Furthermore, KEGG pathway enrichment analysis showed that Wnt signaling pathways were significantly enriched. β-Catenin, a transcriptional coactivator, plays a key role in the canonical Wnt signaling pathway [34]. As the Wnt signaling pathway is activated, β‐catenin is dephosphorylated and reactivated [35]. Accumulating evidence has shown that activating the Wnt/β-catenin signaling pathway can promote the metastatic process in OS and contribute to the induction of EMT by stimulating EMT-inducing transcription factors [36–38]. Usually, β-catenin and E-cadherin exist as E-cadherin/β-catenin complexes in the cell membrane [39]. Moreover, during EMT, degradation of the E‐cadherin/β‐catenin complex results in downregulation of E‐cadherin and accumulation of β-catenin in the nucleus, which enhances the metastatic capacity of cancer cells [40, 41]. In addition, a decreased β‐catenin level can attenuate EMT and inhibit cancer cell migration and invasion [42]. Previous research has revealed that CCR9 can activate the Wnt/β-catenin signaling pathway to enhance invasiveness and chemoresistance in pancreatic cancer [43]. Therefore, we hypothesized that CCR9 may promote OS metastasis by activating Wnt/β-catenin signaling pathways. Another important finding of our study is that knockdown of CCR9 markedly decreased the active β-catenin and total β-catenin levels in OS cells. Overexpression of CCR9 can accelerate OS cell migration and invasion. However, the addition of a Wnt signaling pathway inhibitor (XAV-939) to OE-CCR9 cells abolished this acceleration. Finally, considering the above findings, we believe that CCR9 can activate Wnt/β-catenin signaling pathways to promote OS cell migration and invasion and induce EMT.

In conclusion, the above results confirmed that CCR9 can promote cell migration and invasion and induce EMT by activating the Wnt/β-catenin signaling pathways in OS cells. Therefore, our findings suggest that CCR9 is a promising therapeutic target to inhibit the metastasis of OS and can serve as a novel prognostic marker, as it does in other cancers.

## Supplementary Information

**Additional file 1: Table S1. **The primer sequences used in this study.

## Data Availability

Data sharing is not applicable for this article, as no datasets were generated or analyzed during the current study.

## Abbreviations

OS: Osteosarcoma
TCGA: The Cancer Genome Atlas
EMT: Epithelial–mesenchymal transition
MMPs: Metalloproteinases
ECM: Extracellular matrix
DEGs: Differentially expressed genes
PPI: Protein–protein interaction
GO: Gene Ontology
KEGG: Kyoto Encyclopedia of Genes and Genomes
DAVID: Database for Annotation, Visualization and Integrated Discovery
STRING: Search Tool for the Retrieval of Interacting Genes/Proteins
GSEA: Gene set enrichment analysis
BM-MSCs: Bone marrow-derived mesenchymal stem cells
ATCC: American Type Culture Collection
siRNA: Specific small interfering RNA
PMSF: Phenylmethylsulfonyl fluoride
RIPA: Radioimmunoprecipitation assay
IHC: Immunohistochemistry
CCK8: Cell counting kit-8
BP: Biological process
MF: Molecular function
CC: Cellular component
