# Correction to: CCR9 initiates epithelial–mesenchymal transition by activating Wnt/β-catenin pathways to promote osteosarcoma metastasis

**DOI:** 10.1186/s12935-022-02569-z

**Published:** 2022-04-13

**Authors:** Haoran Kong, Wenhui Yu, Zhuning Chen, Haonan Li, Guiwen Ye, Jiacong Hong, Zhongyu Xie, Keng Chen, Yanfeng Wu, Huiyong Shen

**Affiliations:** 1 Department of Orthopedics, Sun Yat-Sen Memorial Hospital, Sun Yat-Sen University, Guangzhou, People’s Republic of China; 2 Department of Orthopedics, The Eighth Affiliated Hospital, Sun Yat-Sen University, No. 3025, Shennan Middle Road, Futian District, Shenzhen, Guangdong 518033 People’s Republic of China; 3 Center for Biotherapy, The Eighth Affiliated Hospital, Sun Yat-Sen University, No. 3025, Shennan Middle Road, Futian District, Shenzhen, Guangdong 518033 People’s Republic of China

## Correction to: Cancer Cell International (2021) 21:648 https://doi.org/10.1186/s12935-021-02320-0

In this article [1], the annotation was wrong in Fig. 5B and in Fig. 6C, the figure of HOS cells treated by OE-CCR9 + XAV 939 at 0 h was wrong. The revised Figure 5 and its legend and Figure 6 are given below.

**Fig. 5:**
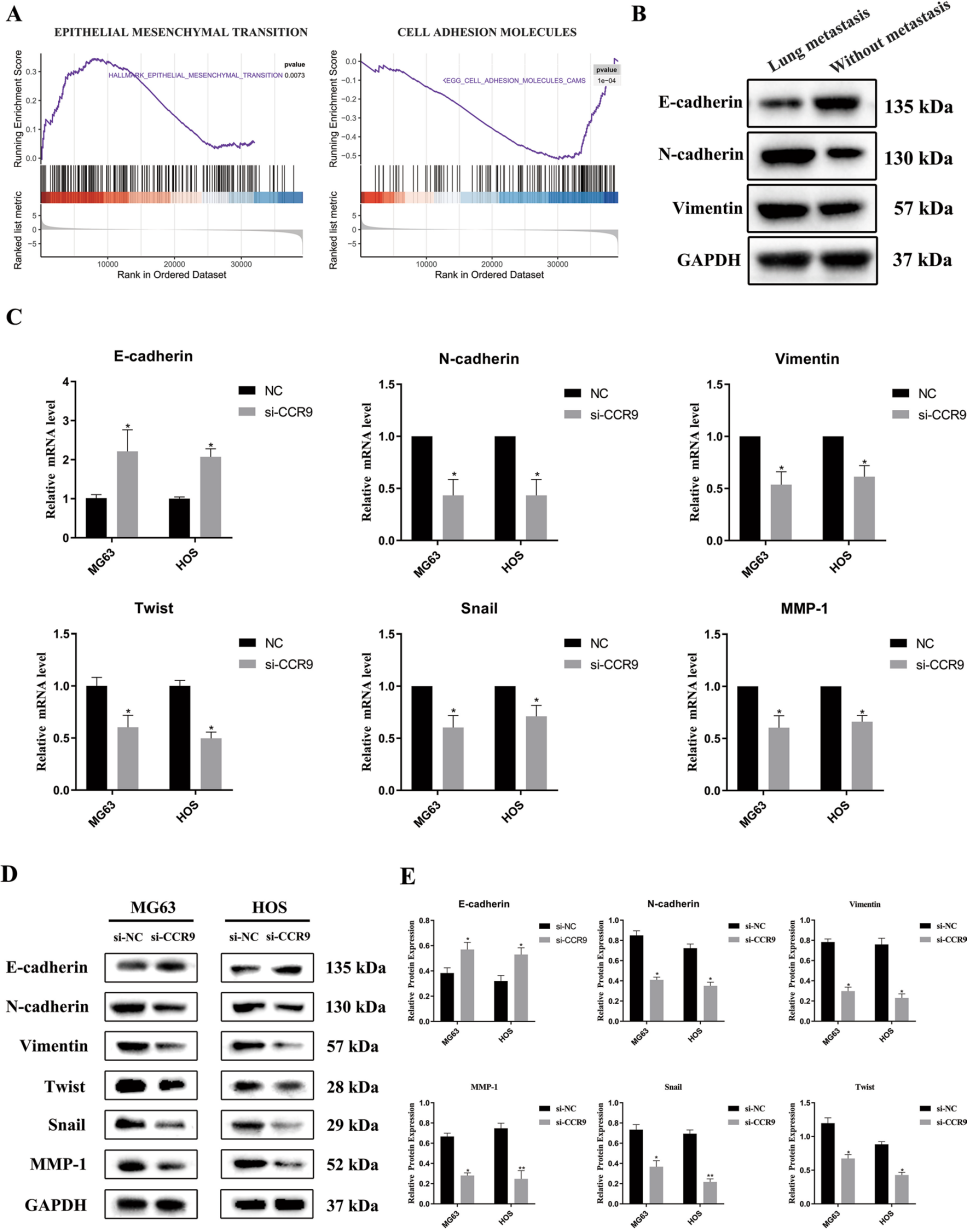
**a** The GSEA results showed significant enrichment of the gene signature associated with EMT and cell adhesion molecules. **b** The protein expression level of E-cadherin was lower in OS tissues with lung metastasis, and the expression of N-cadherin and Vimentin was upregulated. **c** The expression N-cadherin, vimentin, twist, snail and MMP-1, was obviously downregulated, and E-cadherin expression was significantly upregulated in si-CCR9 group. **d** The protein expression levels of EMT-related markers in MG63 and HOS cells. **e** Quantitative data for the protein expression levels of EMT-related markers. Mean ± SD from three independent experiments. *P < 0.05; **P < 0.01; ***P < 0.001

**Fig. 6:**
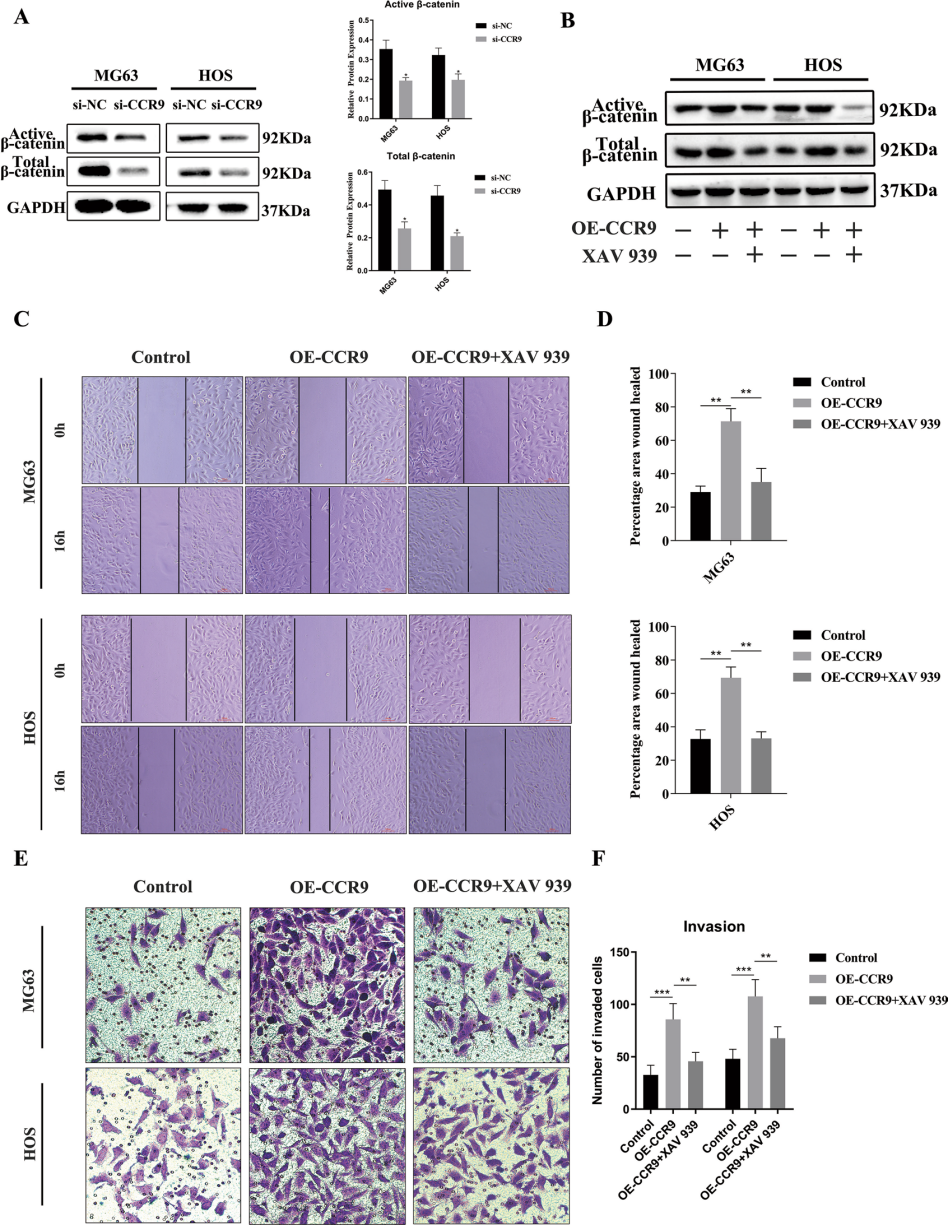
**a** The protein expression levels of active β-catenin and total β-catenin were downregulated in si-CCR9 group. **b** The protein expression levels and quantitative data of active β-catenin and total β-catenin in MG63 and HOS cells were shown. Overexpression of CCR9 activated the Wnt/β-catenin pathway, and the Wnt signaling inhibitor XAV-939 counteracted the activation. **c** Cell migration assay of MG63 and HOS cells. Overexpression of CCR9 increased the wound healing rate and the inhibitor of XAV-939 inhibited this effect. **d** Quantification of the area percentage of wound healing. **e** Transwell invasion assay of MG63 and HOS cells. Overexpression of CCR9 promoted the invasion ability and the inhibitor of XAV-939 inhibited this effect. **f** Quantitative results of the Transwell migration and invasion assays. Mean ± SD from three independent experiments. *P < 0.05; **P < 0.01; ***P < 0.001
