# Supplementary material for: CCR9 initiates epithelial–mesenchymal transition by activating Wnt/β-catenin pathways to promote osteosarcoma metastasis
Source: Cancer Cell Int. 2021 Dec 4;21:648. doi: 10.1186/s12935-021-02320-0 (PMC8642956; doi:10.1186/s12935-021-02320-0)
Supplement: Supplementary file 1 — Additional file 1: Table S1. The primer sequences used in this study. [file 12935_2021_2320_MOESM1_ESM.docx]

**Additional file 1: Table S1. The primer sequences used in this study**

| **Gene** | **Primer** | **Sequence (5′-3′)** |
| --- | --- | --- |
| CCR9 | forward | ATGTCAGGCAGTTTGCGAG |
|  | reverse | TGCAGTACCAGTAGACAAGGAT |
| GAPDH | forward | AATCCCATCACCATCTTCCA |
|  | reverse | TGGACTCCACGACGTACTCA |
| E-cadherin | forward | CTTTGACGCCGAGAGCTACA |
|  | reverse | TTTGAATCGGGTGTCGAGGG |
| N-cadherin | forward | GGGTGGAGGAGAAGAAGACCAG |
|  | reverse | GGCATCAGGCTCCACAGT |
| Vimentin | forward | GGACCAGCTAACCAACGACA |
|  | reverse | AAGGTCAAGACGTGCCAGAG |
| Twist | forward | TCTACCAGGTCCTCCAGAGC |
|  | reverse | CTCCATCCTCCAGACCGAGA |
| Snail | forward | CGGAAGCCTAACTACAGCGA |
|  | reverse | GCCAGGACAGAGTCCCAGAT |
| MMP-1 | forward | AAAATTACACGCCAGATTTGCC |
|  | reverse | GGTGTGACATTACTCCAGAGTTG |
|  |  |  |
